# A Policy-Ready Public Health Guidebook of Strategies and Indicators to Promote Financial Well-Being and Address Financial Strain in Response to COVID-19

**DOI:** 10.5888/pcd20.220209

**Published:** 2023-02-23

**Authors:** Candace IJ Nykiforuk, Ana Paula Belon, Evelyne de Leeuw, Patrick Harris, Lisa Allen-Scott, Kayla Atkey, Nicole M Glenn, Elaine Hyshka, Karla Jaques, Krystyna Kongats, Stephanie Montesanti, Laura M Nieuwendyk, Roman Pabayo, Jane Springett, Aryati Yashadhana

**Affiliations:** 1 Centre for Healthy Communities, School of Public Health, University of Alberta, Edmonton, Alberta, Canada; 2 Centre for Health Equity Training, Research and Evaluation, University of New South Wales, Liverpool, New South Wales, Australia; 3 Ingham Institute for Applied Medical Research and South West Sydney Local Health District, Liverpool Hospital, Liverpool, New South Wales, Australia; 4 Provincial Population and Public Health, Alberta Health Services, Calgary, Alberta, Canada

## Abstract

**Introduction:**

The COVID-19 pandemic has adversely affected the financial well-being of populations globally, escalating concerns about links with health care and overall well-being. Governments and organizations need to act quickly to protect population health relative to exacerbated financial strain. However, limited practice- and policy-relevant resources are available to guide action, particularly from a public health perspective, that is, targeting equity, social determinants of health, and health-in-all policies. Our study aimed to create a public health guidebook of strategies and indicators for multisectoral action on financial well-being and financial strain by decision makers in high-income contexts.

**Methods:**

We used a multimethod approach to create the guidebook. We conducted a targeted review of existing theoretical and conceptual work on financial well-being and strain. By using rapid review methodology informed by principles of realist review, we collected data from academic and practice-based sources evaluating financial well-being or financial strain initiatives. We performed a critical review of these sources. We engaged our research–practice team and government and nongovernment partners and participants in Canada and Australia for guidance to strengthen the tool for policy and practice.

**Results:**

The guidebook presents 62 targets, 140 evidence-informed strategies, and a sample of process and outcome indicators.

**Conclusion:**

The guidebook supports action on the root causes of poor financial well-being and financial strain. It addresses a gap in the academic literature around relevant public health strategies to promote financial well-being and reduce financial strain. Community organizations, nonprofit organizations, and governments in high-income countries can use the guidebook to direct initiative design, implementation, and assessment.

**SUMMARY**

**What is already known on this topic?**

Financial strain is associated with poor physical and mental health outcomes. Most financial strain–related programs focus on behavioral change and, therefore, produce short-term effects.

**What is added by this report?**

Our study introduces a guidebook of evidence-informed strategies for governments and organizations to address the determinants of financial strain. It also provides sample indicators to support assessment of policies and programs.

**What are the implications for public health practice?**

The guidebook offers a road map for decision makers, alone or in partnership with other actors, to design and implement sustainable initiatives for long-term financial well-being.

## Introduction

Austerity policies imposed over recent decades have worsened poverty and living conditions among various populations. With the economic market collapse, loss of livelihoods, and increased caring duties resulting from the COVID-19 pandemic, many people now face unprecedented levels of financial strain and poor financial well-being (1). The latest data from a Canadian survey showed that 57% of households reported deterioration of their financial situation because of the COVID-19 recession and its resultant job loss, temporary layoffs, and reduction in regular paid hours (1). The survey also showed that 48% of households with savings had sufficient resources to cover expenses for 3 months (vs 64% prepandemic), 28% of Canadians said they were short on money at the end of month (vs 19% prepandemic), and 3 in 4 Canadians reported increased stress from financial strain since the beginning of pandemic (1).

Financial strain is different from poverty. Poverty is an objective measure that captures a lack of basic needs (2). Financial strain (also referred as financial hardship, economic stress) (3) occurs when the financial discretionary and nondiscretionary expenditures of a person or household start to exceed their income to a degree that psychologically threatens their intimate relationships and self-esteem (3,4). Financial strain describes people’s current subjective experiences (3) and is an aspect of financial well-being (5). In turn, financial well-being includes objective and subjective measures of current and expected future financial circumstances (eg, economic security based on savings) (4,6). Neither concept is limited to the ability to afford basic needs; they include discretionary spending as part of the emphasis on freedom of choice (4,7–9). Recent resources exemplify how these multidimensional constructs have been measured (5,7).

Financial strain threatens people’s quality of life and health. Adults under financial strain are at higher risk of work absenteeism (10), depression, anxiety, and poor physical health (11–16). Their children are prone to loneliness, depression, other mental health issues, and disability into adulthood (12,16). Financial strain leads to poor health, and poor health leads to financial strain. Financial strain disproportionately affects equity-seeking groups including women, Indigenous peoples, seniors, and immigrants (3,17–19). People who have greater privileges (eg, property ownership, stable income) can also experience financial strain (20) related to life-course events (eg, having children, retirement) (7) or to unexpected events (eg, illness) (13).

To support populations in greater need, governments and organizations have implemented financial strain–related initiatives (21,22), for example, through income support. However, such programs and services offer only temporary relief, as observed during the global financial crisis caused by the pandemic. Greater synergy between government and nongovernment organizations is needed to address the root causes of financial strain and promote financial well-being following the pandemic. A gap exists between the use of current evidence and coordinated, equitable, system-level implementation (23). This article responds to this gap by presenting a public health guidebook of strategies and indicators (hereinafter Guidebook) for action on financial well-being and financial strain for use by policy makers and public health practitioners in high-income contexts (24). 

**Figure 1:**
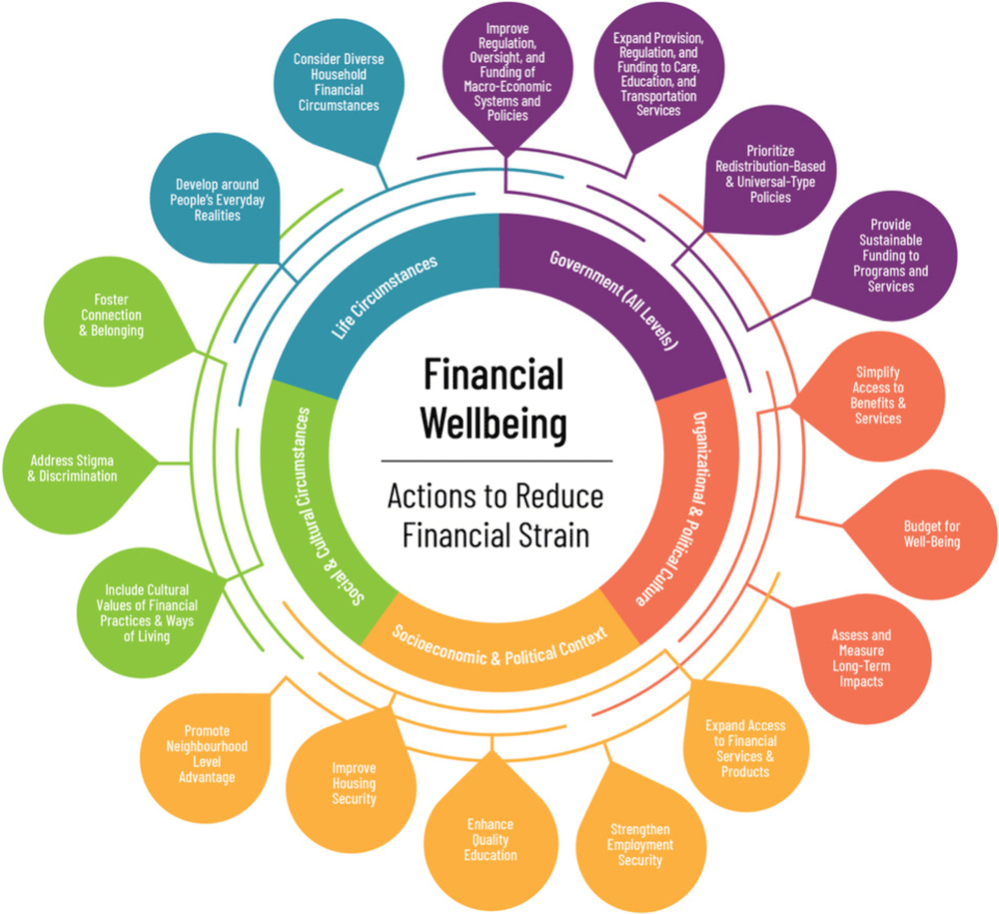
Action-oriented public health framework on financial well-being and financial strain. Reprinted with permission from: *Action-oriented Public Health Framework on Financial Wellbeing and Financial Strain: Executive Summary *(25).

Our Guidebook expands the operationalization of an action-oriented public health framework (hereinafter framework) we previously developed (25), which indicates 17 entry points for action grouped into 5 domains (Figure 1). Except for government at all levels, the other 4 domains are applicable to both governments and nongovernment organizations. The main goals of the Guidebook are to 1) strengthen the evidence base to support the design and implementation of more effective, equity-informed initiatives on any topic related to financial strain and financial well-being (eg, financial empowerment, employment supports) for various audiences (eg, 2SLGBTQIA+ [two-spirit, lesbian, gay, bisexual, transgender, queer or questioning, intersex, asexual, and additional sexual orientations and gender identities] communities, Indigenous peoples); 2) increase the evaluative capacity of governments and organizations in monitoring outcomes of their initiatives; 3) encourage reflection on whole-of-society contributions from other actors who share the same goals to improve understanding of the interconnectedness of actions; 4) support creating or strengthening partnerships between public, private, and nonprofit sectors; and 5) promote meaningful collaboration with community partners for enhanced relevance and cultural sensitiveness of initiatives.

## Methods

**Figure 2:**
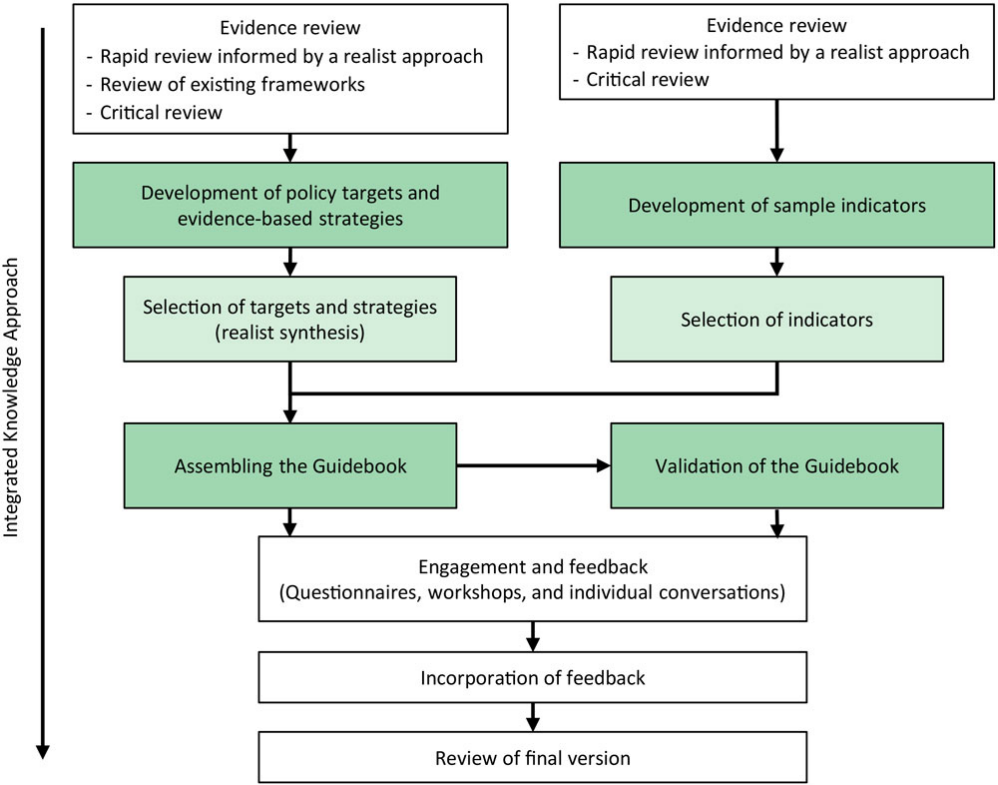
Integrated knowledge approach showing the 4 stages in the development of a policy-ready public health guidebook of strategies and indicators to promote financial well-being and address financial strain in response to the COVID-19 pandemic.

Our multimethod research–practice project, conducted in Canada and Australia, adopted an integrated knowledge translation process to enhance practical relevance and applicability of the Guidebook (Figure 2). We used a variety of methods to design the Guidebook (24).

### Integrated knowledge translation

An integrated knowledge translation approach informed the refinement and validation stages of the Guidebook. To ensure comprehensiveness, contextual appropriateness, cultural sensitivity, and practical relevance and to enhance usability of the resource in various practice settings, we engaged with practitioners, decision makers, policy makers, and researchers to incorporate academic and practice expertise and experiential knowledge. We first conducted a scan of Canadian and Australian national nonprofit organizations and government agencies directly or indirectly involved in the promotion of financial well-being or reduction of financial strain. To achieve professional and geographic diversity, we categorized the organizations according to location and sector. After reviewing the list for representation of diverse organizations, we performed a stakeholder analysis to purposively identify representatives of the selected government agencies and civil society in Canada and Australia as described above. The representatives were any staff member who was involved in a professional capacity with actions related to financial strain and financial well-being. By using available contact information from the organizations’ websites, we contacted each representative by email with an invitation letter. We asked representatives to forward the message to another member of their organization if they were unavailable to participate themselves. Participants were not asked to share any lived experiences with financial strain or with seeking help through support systems, although participants’ own lived experiences may have shaped their participation.

In Canada, 16 members affiliated with government (eg, financial sector, public health agencies), research centers, universities, and nonprofit organizations (eg, groups supporting Black female entrepreneurs, Indigenous communities, and women, girls and gender-diverse groups in the justice system) accepted our invitation and became our practice advisory committee (PAC). In Australia, only 6 representatives of organizations participated in the project despite extensive recruitment efforts; therefore, a PAC was not formed in Australia. Participants in both countries held positions as scientific directors, chief executive officers, project leads, research and policy analysts, and program coordinators. To support meaningful engagement of the participants and accommodate their needs, we employed different methods to collect their feedback: workshops, individual meetings, and online questionnaires.

Both the University of Alberta Research Ethics Board (Pro00102631) and the University of New South Wales Human Research Ethics Committee (HC200896) provided ethics approval to record written and verbal information and to use the data gathered through our workshops, surveys, and one-on-one conversations. All participants gave implied informed consent by proceeding with our data collection activities.

### Targets and strategies

Through integrated knowledge translation activities, participants reached consensus about the need to indicate where to act and what to do for each of the 17 entry points for action in the framework. Participant feedback led to the development of targets and evidence-informed strategies to compose the Guidebook. We identified targets and strategies through the review of findings from a rapid review informed by a realist approach (26–29) and assessment of existing frameworks related to financial well-being and financial strain (Figure 2). These 2 methods were previously used in the methodology to develop the framework’s entry points for action (25).

Rapid review identified peer-reviewed academic and practice-based literature reporting community-led and government initiatives in high-income countries (30) that directly or indirectly aimed to promote financial well-being. We used Preferred Reporting Items for Systematic Reviews and Meta-Analyses (PRISMA) guidelines (31). We searched peer-reviewed resources published from 2015 through 2022 in MEDLINE, PsycINFO, and Web of Science (Social Science Citation Index) by using terms related to financial strain and financial well-being and initiative. We used ProQuest (ProQuest LLC) and Informit (Pearson Education, Informit) to identify practice-based resources and filtered the results by source type (eg, reports). Additionally, we performed a targeted search in Google Advanced (Google LLC) by using a modified version of the search terms and limitations from the search of peer-reviewed literature. With a predefined set of inclusion and exclusion criteria, 2 independent reviewers (A.P.B. and N.M.G.) each screened 50% of 3,516 peer-reviewed resources retrieved. A single reviewer (K.J.) assessed the 6,035 practice-based resources; a second reviewer (A.Y.) screened a 10% sample to minimize bias. The Mixed Methods Appraisal Tool (MMAT) (32) was applied for quality assessment of peer-reviewed literature on empirical research. We extracted data using EPPI-Reviewer software (EPPI Centre). We used equity principles to organize initiatives by level of intervention, reach, and impact (eg, universal, targeted) (33). To analyze the 39 peer-reviewed articles and 36 practice-based resources included in the review, we employed a realist approach (26–29) to identify underlying, contextual factors of intended and unintended outcomes and equity considerations. We applied a context-mechanism-outcome configuration to reveal what works for whom and in what circumstances. Through a collaborative data analysis process, we discussed the findings and used Miro (a visual collaboration platform; https://miro.com/) to map context-mechanism-outcome relationships. More information on the rapid review is published elsewhere (30).

We also identified 14 frameworks (4–8,34–42) on financial strain and financial well-being through a purposive literature search. By using social determinants of health (43,44), health equity (45–47), and health-in-all policy lenses (48,49), we developed a form to identify main characteristics, theoretical perspectives, and structural determinants and to assess strengths and limitations of these frameworks, which came from economic anthropology, happiness research, financial services, consumer research, and business, commerce, and marketing. Of note, only 1 framework came from public health (41) — a notable gap in the literature. In defining financial health as a function of saving, spending, borrowing, and planning activities, the framework was limited in its ability to articulate it as a social determinant of energy, housing, and food security.

We performed a critical review of academic and practice-based literature to identify further targets and strategies aligned with the 17 entry points of action in the Framework. We applied realist synthesis lenses (50) to select the most contextually relevant and appropriate targets and strategies to be included in the Guidebook. Feedback from the authors and participants through our workshops supported the development of targets and strategies.

### Process and outcome indicators

We developed sample indicators to illustrate specific ways that organizations and governments could assess the progress and outcome effects of their initiatives (Figure 2). In the data extraction phase of the rapid review, we systematically identified indicators from the included resources. We documented indicators that were specific to different equity-seeking groups (eg, Black, Indigenous, youth, women, and immigrants). By using a data extraction template, we collected quantitative or qualitative, process, or outcome indicators from the main text, footnotes, figures, tables, and references of the academic and practice-based literature reviewed. We then created a summary table with all potentially relevant indicators. Indicators were then matched with the evidence-informed strategies.

To ensure a more diverse set of indicators to meet the needs of a wide range of initiatives and to address a lack of indicators for some strategies, we also reviewed resources included in the literature review and searched additional materials. We then further refined indicators to ensure equity focus and encourage disaggregated analysis by different social locations (eg, through social stratification).

### Assembling, refining, and validating the Guidebook

The Guidebook comprises selected targets, evidence-informed strategies, and sample indicators related to the framework’s corresponding entry points for action. To inform refinement and validation of the Guidebook, we invited the research team, the Canadian PAC, and Australian representatives of community organizations to provide critical feedback on how to make it more feasible and relevant to community members, practitioners, policy makers, and researchers in Canada, Australia, and other high-income countries (Figure 2). They were asked to complete an online questionnaire and participate in a workshop or individual conversations, depending on their needs. This flexibility was necessary because our research began in the first year of the pandemic. The engagement activities occurred from September 2020 through May 2021.

We designed our questionnaire in the REDCap (Research electronic data capture) system (51). It was divided into the 5 domains and the 17 entry points for action of the framework, under which the corresponding targets and strategies were listed. In the evaluation of each target and strategy, we asked the following questions: “How can we strengthen the Guidebook? Please let us know how we can clarify the information and make the Guidebook more practice-oriented and user-friendly.” We set response categories to allow a single answer: clear or unclear. When unclear was marked, a pop-up question appeared to collect specific feedback and suggestions for improvement. At the end of each domain, we asked for any additional feedback, concerns, or suggestions for that specific domain. Our last multipart reflection question was “Based on your work experience and expertise, what is the relevance of these strategies to your practice? How might you use the strategies?” Research team members as well as the Canadian PAC received a REDCap link by email 1 week before the workshop; they had 1 week to complete the survey. The overall response rate for the survey was 45.4%.

We then held one Zoom (Zoom Video Communications, Inc) workshop each with the Canadian PAC and the research team to gather their collective feedback and support consensus building in a group setting, building on participants’ reflections from the survey. For the PAC workshop, we had 3 breakout rooms, given the high number of participants. Research staff in each breakout room provided some guidance to the discussion (eg, asking about what changes would make the Guidebook more practice-oriented and user-friendly) and took notes to report back at the large discussion session. Notes were shared with the entire group via Miro. For the research team workshop, we had a large group discussion, and L.M.N. took notes in Miro.

To respect the limited time available of the 6 Australian participants, we used a modified version of the survey questionnaire to collect their individual feedback in a one-on-one conversation. The anonymous, collective feedback was then used to revise the contents and formatting of the Guidebook. Feedback from the Canadian PAC and Australian participants improved clarity and enhanced relevance and usability of the Guidebook for diverse users.

## Results

**Figure 3:**
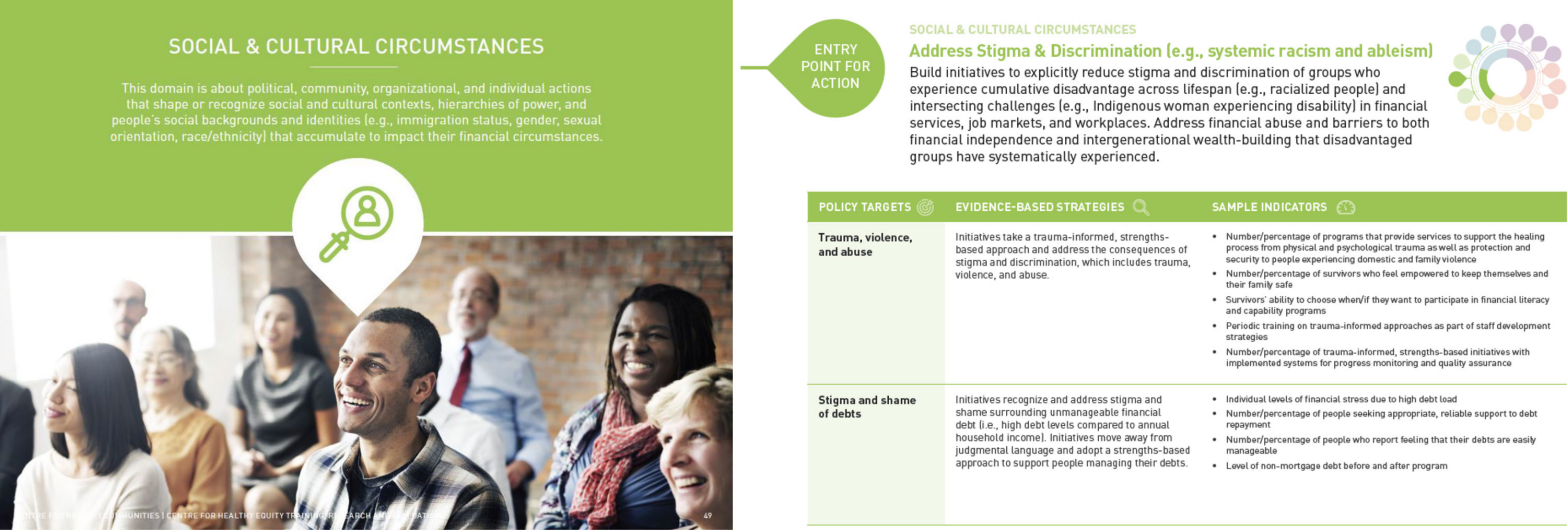
Sample pages from the *Guidebook of Strategies and Indicators* *for Action on Financial Wellbeing & Financial Strain* (24), a policy-ready public health guidebook of strategies and indicators to promote financial well-being and address financial strain in response to COVID-19. Reprinted with permission.

The Guidebook represents the culmination of the various stages of our multimethod research project. The final product (24) is a concise, yet substantial and comprehensive set of evidence-informed strategies and indicators to inform development, implementation, and evaluation of financial well-being initiatives (Figure 3). In total, the Guidebook presents 62 policy targets and 140 evidence-informed strategies, which correspond to the 17 entry points for action described in the framework. The list of indicators is not exhaustive; instead, the indicators are examples that can be used to support further brainstorming in the development or selection of other indicators specific to setting or population. English and French electronic versions of the Guidebook are available online on the Centre for Healthy Communities website (uab.ca/chc) and Centre for Health Equity Training Research and Evaluation website (https://chetre.org/).

### Using the Guidebook

**Figure 4:**
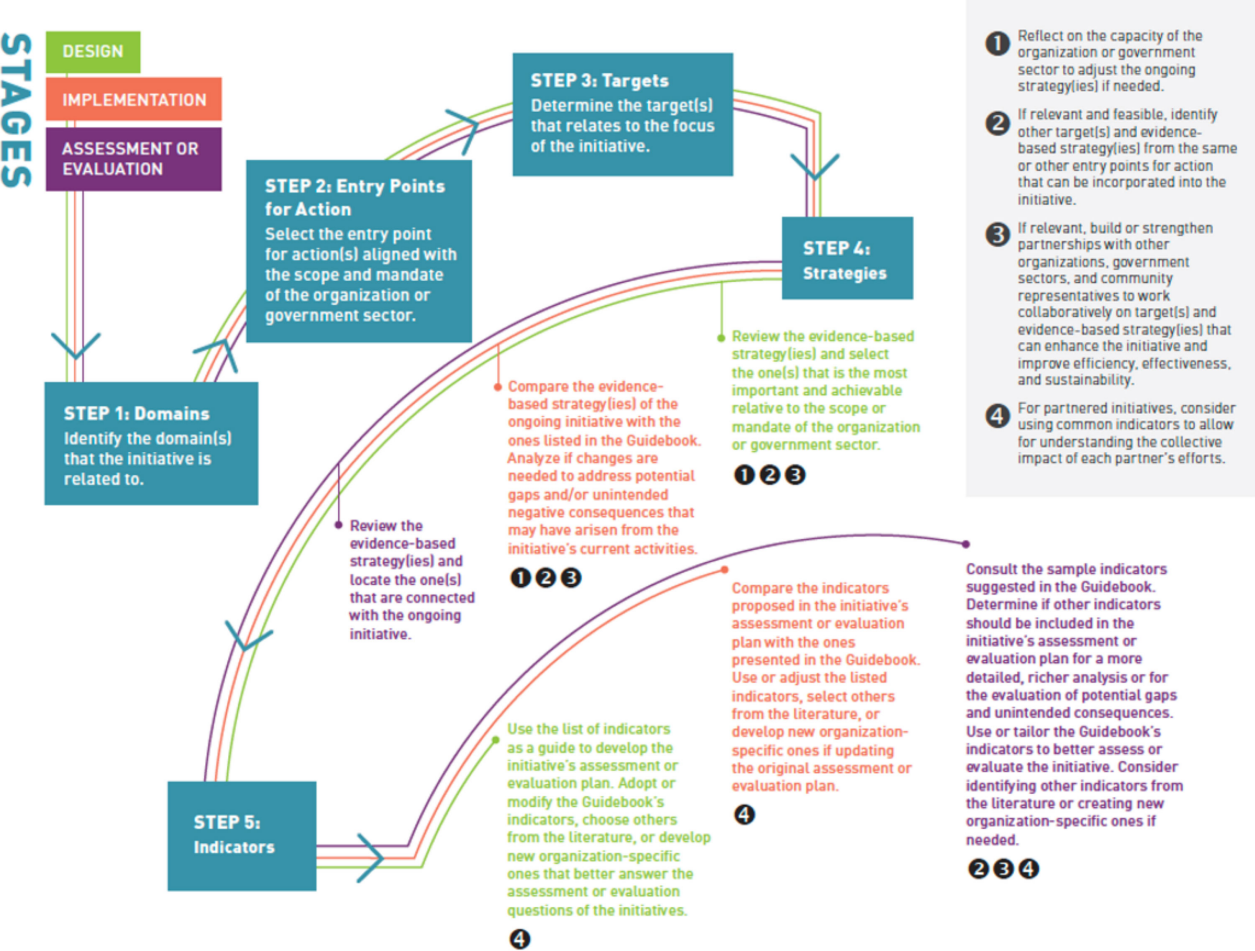
Flowchart providing guidance for using the *Guidebook of Strategies and Indicators for Action on Financial Wellbeing & Financial Strain* (24). Reprinted with permission.

To provide guidance on how to use the Guidebook, we developed a flowchart that is divided into stages of initiatives and steps. Stages of initiatives are design, implementation, and assessment (Figure 4). Use of the Guidebook starts with identification of the stage of the initiative. Next, the user follows the colored lines throughout the flowchart to identify the steps to be taken. The first 3 steps are about identifying domains, entry points for action, and targets. Steps 4 and 5 present prompts to support identification of strategies and indicators, respectively. Governments and organizations are invited to reflect on what additional entry points, targets, and strategies may further meet their needs and to combine those components. Indicators in the Guidebook are only examples; governments and organizations are encouraged to select or develop new ones that may better respond to their needs. Social stratification in the measurement of process and outcomes is highly recommended and some indicators are disaggregated by income, sex and gender, and other factors for illustrative purposes.

### Audience

Our Guidebook was designed for researchers, practitioners, decision makers, and policy makers in Canada, Australia, and other high-income countries. The Guidebook’s strategies and indicators can be applied and adapted to the context and dynamics of each high-income country. Researchers can use the set of strategies when designing initiatives and apply the indicators in the Guidebook to evaluate their own initiatives or those led by other organizations and governments. For end users, the Guidebook facilitates the identification of high-impact strategies to reduce financial strain and improve financial well-being, thereby promoting health and equity. The Guidebook also presents indicators to support implementation evaluation and impact assessment. The Guidebook is applicable to the different contexts and mandates of private, public, and nonprofit organizations and to municipal, provincial, territorial, state, and federal governments. It can inform downstream, midstream, and upstream policies, programs, and services that directly or indirectly contribute to better outcomes in financial well-being, such as strategies to address systemic racism in the labor market, the housing affordability crisis, or issues related to expensive and low-quality, long-term care services. It can also inform actions targeting the unique needs of equity-seeking groups, such as women facing family violence or the elderly without retirement savings.

## Discussion

We have introduced a comprehensive, action-oriented, public health Guidebook of strategies and indicators for organizations and governments in high-income countries working directly or indirectly on reducing financial strain and promoting financial well-being. The Guidebook provides a comprehensive set of evidence-informed strategies and process and outcome indicators to inform design, implementation, and assessment of financial well-being and initiatives related to financial strain. The Guidebook is the first of its kind. It integrates the literature on social determinants of health (43,44), health equity (45–47), and health-in-all policies (48,49) in the context of financial strain and financial well-being. In so doing, it de-emphasizes individual-level interventions that usually target financial behaviors; instead, it presents actions that governments and organizations can take to address the structural factors of financial strain and financial well-being. The Guidebook is an action-oriented resource that is meant to inform policies, programs, and services. It provides evidence-informed strategies for system-based solutions.

The Guidebook was developed under a rapid-response funding opportunity in response to the COVID-19 pandemic, which exacerbated financial strain. However, its utility is not limited to the pandemic period. It was designed for flexible use across various contexts to mitigate and prevent ongoing negative health consequences of enduring financial strain, or it can be applied in future global shocks such as another pandemic, environmental disaster, or economic recession.

The strategies and indicators commonly used in the literature to address and measure financial strain and poor financial well-being are narrow and overwhelmingly represent the concepts of behavioral economics (52). Such concepts, which are increasingly popular in social policy, economics, and psychology, have often been framed around the idea of individual financial decision making, emphasizing the individual’s behavior and responsibility for making rational decisions regarding the acquisition and consumption of resources. Very little attention has been paid to social determinants and other structural or system drivers of financial strain and well-being that are outside the individual’s ability to influence or control. Similarly, social and health inequities are not often examined in the context of the system drivers of financial strain and financial well-being. Also, little information is available on strategies for action and indicators of success to address and assess the long-term health effects of poor financial well-being and financial strain. The Guidebook addressed this gap in knowledge and practice by supporting public health practitioners, decision makers, policy makers, and researchers in high-income countries with initiative design, implementation, and assessment. The Guidebook presents a list of evidence-based high-impact actions that are more likely to have long-term, positive effects on people’s financial circumstances, particularly for equity-seeking groups.

### Limitations

Our project had limitations. The Guidebook was developed under a rapid project process and originated within a framework that covered a wide range of areas. As such, we conducted a high-level review of the large amount of available evidence. We also recognize that the literature review we conducted to support the development of the strategies and indicators was not exhaustive and may not have captured all relevant literature in each topic area. To address both methodologic limitations, we combined multiple methods to collect and analyze academic and practice-based evidence and engaged with an international, interdisciplinary group of academics, practitioners, and representatives of governments that provided critical feedback on the items included in the Guidebook. PAC members in Canada and stakeholders in Australia were purposively selected to represent a broad, diverse body of policy and technical expertise. Their extensive knowledge helped select a variety of appropriate and relevant policy targets and strategies alongside technically sound, feasible, and meaningful indicators that are responsive to user needs.

Additionally, rapid review methodology with realist components is being employed increasingly in evaluation and implementation research (53,54). However, we found few examples of its use for the development of a guidebook (53,55). As such, this project represents a novel approach to evidence synthesis and translating knowledge into action that may guide future research–practice collaborations.

### Conclusion

Greater synergy between public health and government, community, and financial sectors is needed to assess the impact of financial strain and to design and implement financial well-being initiatives. Our multimethod collaborative research project represents the collective efforts of members of academia, government, and nonprofit organizations to develop, within a short timeframe and at the height of the pandemic, a relevant and useful tool to guide action on financial strain and financial well-being over the short and long term. The Guidebook is a key step in addressing the significant existing gap in public health evidence and action-oriented resources to promote financial well-being and reduce financial strain for all populations, but particularly for equity-seeking groups. The actionable areas presented in the Guidebook can inform initiatives that have the potential to achieve sustained, positive progress in securing financial well-being in the long term and to promote equitable conditions for all people to enjoy financial security. The Guidebook delineates the complex strategies of equitable, systems-oriented action needed to address financial strain and financial well-being through coordinated, transdisciplinary, multidisciplinary initiatives.

In our efforts to address uptake barriers (eg, language, knowledge, skills) and improve reach to different target audiences (56), we will continue to partner with government sectors and professional and community organizations and to attend scientific and practice-based events to share the Guidebook widely. The Guidebook is a living document. Feedback provided by end users will be reviewed and, where appropriate, incorporated in future versions of the Guidebook. To optimize dissemination of the Guidebook, our next step will be to create a user-friendly, interactive, web-based version to support end-users’ nonlinear, more flexible interaction with the Guidebook.
